# Tackle Risk Factors for Head Injury Assessments (HIAs) in Sub-Elite Rugby League and Recommendations for Prevention: Head Contacts from Upright Tackles Increase the HIA Risk to Both Ball Carrier and Tackler

**DOI:** 10.1186/s40798-024-00696-7

**Published:** 2024-04-17

**Authors:** Martin A. Lang, Ross Tucker, Suzi Edwards, Grant L. Iverson, Andrew J. Gardner

**Affiliations:** 1 School of Medicine and Public Health, College of Health, Medicine and Wellbeing, University of Newcastle, Callaghan, NSW Australia; 2 Institute of Sport and Exercise Medicine, Department of Sport Science, University of Stellenbosch, Stellenbosch, South Africa; 3 Sydney School of Health Sciences, Faculty of Medicine and Health, The University of Sydney, Camperdown, NSW Australia; 4 Department of Physical Medicine and Rehabilitation, Harvard Medical School, Boston, MA USA; 5 Mass General for Children Sports Concussion Program, Boston, MA USA; 6 Department of Physical Medicine and Rehabilitation, Spaulding Rehabilitation Hospital, Schoen Adams Research Institute at Spaulding Rehabilitation, Charlestown, MA USA

**Keywords:** Head injury assessment, Mild traumatic brain injury, Head impact events, Tackle events, Head injury prevention

## Abstract

**Background:**

The rugby league tackle has been identified as the game event with the greatest propensity for a clinically diagnosed concussion. This study aims to replicate the work conducted in professional rugby league and rugby union by examining Head Injury Assessment (HIA) events to determine the associated tackle characteristics that increase concussion risk in sub-elite rugby league players. This comparison between competition levels is important due to the less developed physiological and tackle proficiency characteristics of sub-elite rugby league players and the fewer resources available for an on-field diagnosis, compared to the elite level of the sport.

**Results:**

Tackles resulting in Head Injury Assessments (HIAs, *n* = 131) and 2,088 tackles that did not result in a head injury were identified and coded from one season of the 2019 Queensland Cup. The body position of both ball carrier and tackler, tackle height, and body contact areas were evaluated. The propensity for tacklers to undergo a head injury assessment was 1.49 HIAs per 1,000 tackles, equating to a 2.5-fold higher risk than that of the ball carrier (0.59 HIAs per 1,000 tackles). The risk for an HIA was 2.75-fold greater when the tackler was upright (2.89 HIAs per 1,000 tackles) compared to a bent-at-the-waist tackler (1.05 HIAs per 1,000 tackles). The greatest risk for the tackler and ball carrier sustaining an HIA occurred when the tackle height was high, with head-to-head contact having the greatest propensity for an HIA (44.37 HIAs per 1,000 tackles). HIA risk was also greater for both players when the ball carrier did not employ an evasion strategy (3.73 HIAs per 1,000 tackles).

**Conclusions:**

The study replicates results from research in elite rugby league and rugby union. A combination of higher head contact/proximity and upright body position significantly increase an HIA risk. Tackler head position and ball carrier evasion behaviours also affect risk, suggesting that injury prevention strategies designed to reduce tackle height and improve tackle technique by focusing on head position, body position, and in a novel finding, ball carrier evasion, may reduce head injury risk in sub-elite rugby league players.

## Key Points

**•** An upright tackle results in the greatest risk for a Head Injury Assessment (HIA) for both the tackler and ball carrier.
**•** The tackler is more likely to experience an HIA than the ball carrier.
**•** When a ball carrier does not employ an evasion strategy, the risk for an HIA is greater for both the ball carrier and the tackler.

## Background

Rugby league is a full-contact collision sport that involves multiple tackle events per match [1]. A tackle is defined as “any event where one or more tacklers attempt to stop or impede the ball carrier irrespective of whether the ball carrier was brought to the ground” [2]. One of the inherent risks of participation in rugby league is concussion [3, 4], with the tackle identified as the game event leading to the most concussions, and which also has the greatest risk for head impact events (HIEs) and Head Injury Assessments (HIAs) [1]. HIAs are either off-field medical evaluations for concussion, indicated when a significant head impact event with the potential to cause concussion is noted, or the immediate and permanent removal of a player if the player demonstrates any signs or symptoms of concussion, that are evident after head impact.

The tackler is at more risk of an HIE than the ball carrier [1, 3, 5]. In elite men’s rugby league, frequent mechanisms of injury in concussed tacklers include head-to-shoulder contact and head-to-head contact in upper body tackles, and head-to-hip contact in tackles below the torso [1]. An almost identical mechanism of injury has also been reported in elite men’s rugby union, where the HIA and concussion injury propensities are greater for the tackler than for the ball carrier and upright tackles involving proximity and contact of the tackler’s head above the sternum of the ball carrier are more likely to result in an HIA than contact below the sternum during bent at the waist tackles [1, 6]. Variables such as tackle height, direction, and speed of the tackler have also been identified as risk factors for concussion in both rugby codes [7].

It is not known whether the mechanisms of injury during tackle events, identified at the elite levels of rugby league and rugby union, are the same at the sub-elite level of play. It is possible that tackle technique, tackle proficiency [8], conditioning [2], team cohesion and general differences in the way the game is played may influence the relative risk of certain tackle characteristics. These factors may impact the execution of defensive patterns performed by sub-elite players which ultimately may present with different head injury mechanisms compared to elite players. It is important to understand whether these differences exist, so that targeted approaches to risk reduction can be developed. Accordingly, the aim of this study was to replicate previous research in the National Rugby League (NRL) to describe HIA risk factors in a sub-elite rugby league competition (the Queensland Cup, the highest level of state-based club rugby league, one tier below the NRL) [1]. The primary objective of this study was to evaluate tackle-based risk factors for HIAs in sub-elite male rugby league players. Other tackle characteristics that may influence the risk for head impacts including tackle height, player head position, ball carrier evasion methods, and body mass and height differential between players, were also evaluated.

## Methods

### Participants

This case-control study evaluated match footage from the 2019 Queensland Cup season. The QLD Cup is Queensland Rugby League’s (QRL) highest level of club competition. The QRL are the governing body of rugby league in the state of Queensland, Australia. The QLD Cup is a second-tier (sub-elite) competition for the NRL, the elite, national club rugby league competition in Australia. The QRL is predominantly made up of younger players who are contracted to NRL clubs, along with a number of older players who may have NRL experience. It is utilised as a pathway to the NRL [9]. Differing and/or underdeveloped skill levels and physiological characteristics are observed at this level competition [2]. The QLD Cup comprises fourteen teams, competing over 23 rounds, with a four-week finals system between eight qualifying teams. Therefore, during a regular season, there are 161 games (7 pairs of teams, each playing 23 games). The finals (post-regular season) feature 9 games making a total of 170 games in the analysed season. All players gave prior consent to the Rugby League Players Association (RLPA) and the QRL to have their deidentified data (injury) used in research endorsed by the QRL Research Committee. This study was approved by the governing institution human ethics committee and endorsed by the QRL Research committee. The study was conducted in accordance with the Helsinki Declaration and approved by the University of Newcastle Human Ethics Committee (Ref No. H-2012-0344).

### Procedures

All HIEs were identified by the QRL’s medical staff and via the sideline injury surveillance team (sports trainers). An HIE was identified as any event that resulted in a significant impact to a player’s head. An HIA was identified as a head impact event that necessitated either the temporary or permanent removal of a player from the match, who is suspected of sustaining a concussion. In the case of temporary removal, where the head impact has the potential to cause a concussion, the player receives an off-field medical evaluation which guides return to play decisions at the time of injury. Permanent removal occurs when any of the pre-identified signs of concussion are identified by the surveillance teams. This is completed in accordance with the QRL concussion recognition and management protocols [10]. HIA cases, comprising temporary and permanent removals, are reported in the results.

For this study, the focus was on the injured player. When the ball carrier was injured in a tackle involving more than one tackler, the tackler’s actions that caused the HIA were coded. Coding included the elements of technique that could be observed from video footage. These include player body positions for both the tackler and ball carrier at contact, tackle height, location of head contact in injured players, and nearest head-to-opponent’s body proximity of the opponent for the control cases. The tackler’s head position and the evasion strategy performed by the ball carrier were also coded.

Video analysis of HIEs was conducted using the STATS Edge platform, with full access provided by the QRL. The broadcasted games provided multiple camera angles, with one broadcasted game scheduled per week (23 of the 161 regular season games [∼ 14%] and all nine of the finals series games; 32/170 or ∼ 19% of games for the entire season). For the non-broadcasted games, one camera angle was available. The video analysis was conducted by a single author (MAL), utilising a coding matrix including 46 variables for each HIA incident. Many of the variables that were coded have been described previously [1]. The coding matrix was developed using templates utilised in professional rugby union and rugby league [3]. Some key definitions include the tackle, which is defined when progress has been halted by one or more opposing players. Another variable is the ball carrier evasion technique, which defines the technique the ball carrier adopts to avoid being tackled.

A group of control tackles (defined as those tackles that did not result in an HIA for either the ball carrier or the tackler, *n* = 2,087) from six randomly selected games from the 2019 QRL season were coded to allow calculation of the total number of control tackles from a 170 match season, which in turn allowed calculation of the propensity of various tackle characteristics in normal match play, in HIAs per 1,000 tackles. Tackle events were excluded if the video quality did not permit accurate identification of the player and/or the contact area. Any tackle during the six control games that resulted in an HIA were not included in the control tackle data. A total of five tackles resulting in an HIA occurred during one of the six control games.

### Data Analysis

HIA propensity for each tackle characteristic was calculated by dividing the total number of HIA events occurring from that tackle circumstance [2], by the total number of tackle circumstances of that type, obtained from the control sample from the six randomly selected matches which were extrapolated from the 170 matches in the competition [2]. The six randomly selected coded games included 9 different teams, with one game from the first third of the season, two from the middle third, and three from the last third of the season. The relative likelihood of an HIA occurring in a given tackle circumstance was related to other circumstances through calculation of an incident rate ratio, the ratio of two propensities being compared. The probability of each tackle characteristic being associated with an HIA was assessed using a Poisson regression with a log link function, using exposure to the characteristic as the offset variable to compare predictor/independent variables. The 95% CI are shown for propensity and IRR, and significant differences inferred when the 95% CI did not overlap, and when the 95% CI of the Incidence Rate Ratio did not cross 1.0.

## Results

Of the 131 HIAs coded during the 2019 QRL season, 123 (93.9%) occurred in a tackle event with the remaining 8 HIAs (6.1%) observed in game play incidents that did not involve a tackle contest (e.g., an aerial contest or an off-the-ball collision). Overall, the 131 HIAs coded occurred with a propensity of 2.08 (95% CI: 1.75 to 2.47) HIAs per 1,000 tackles. There were 88 HIAs to the tackler (67.2%) with a propensity of 1.49 (95% CI: 1.21 to 1.84) HIAs per 1,000 tackles. Ball carrier HIA (*n* = 35) propensity was 0.59 (95% CI: 0.42 to 0.82) HIAs per 1,000 tackles. In total, 109 HIAs resulted in permanent removal, with 22 HIAs returning to play in the same game. Tacklers were significantly more likely to sustain an HIA than ball carriers (IRR tackler vs. BC 2.51, 95% CI 1.70 to 3.72).

### Tackler Body Position

Table 1 shows the number of HIAs, and calculated propensity for HIAs, for various body positions of the tackler and ball carrier. The table is divided into sections showing HIAs (HIA to either player), and then HIAs to the tackler and the ball carrier as a function of the body position of each player separately.

An upright tackler accounted for 84 HIAs (64.1%), with a propensity of 2.89 (95% CI: 2.33 to 3.58) HIAs per 1,000 upright tackles. In contrast, the bent-at-the-waist tackler accounted for 26 HIAs (19.8%), with a propensity of 1.05 (95% CI: 0.71–1.54) HIAs per 1,000 bent tackles. When considering the tackler only, 55 tackler HIAs (62.5%) occurred when executing an upright tackle, with a propensity of 1.89 (95% CI: 1.45–2.46) HIAs per 1,000 tackles, as opposed to executing a bent-at-the-waist tackle with 23 (HIAs (26.1%) and a propensity 0.93 (95% CI: 0.62–1.40) HIAs per 1,000 tackles. High tackler HIA propensities were also observed for diving (3.27, 95% CI: 1.36–7.86) and leaping (3.78, 95% CI: 1.22–11.72) tacklers, but such cases were rare (5 and 3 HIAs respectively) (Table 1).

The risk of an HIA to the ball carrier was greatest when the tackler was upright (27 HIAs, with a propensity of 0.93 (95% CI: 0.64–1.36) HIAs per 1,000 upright tackles). High propensities were also observed for leaping (3.78, 95% CI: 1.22–11.72) and to a lesser extent flopping (1.10, 95% CI: 0.28–4.40) though these cases were rare (3 and 2 respectively) (Table 1).

**Table 1:** Propensity for tackle-related HIAs as a function of body position, stratified by tackler and ball carrier

	HIA events	Propensity (HIAs per 1,000 events)
Ball carrier	35	0.59 (0.42–0.82)
Tackler	88	1.49 (1.21–1.84)
Neither	8	NA
**Total (Tackler and Ball carrier)**	**123**	**2.08 (1.75–2.47)**
**Tackler body position All HIAs**		
Upright	84	2.89 (2.33–3.58)
Bent at waist	26	1.05 (0.71–1.54)
Diving	7	4.58 (2.18–9.61)
Leaping	6	7.56 (3.40-16.83)
Flopping	4	2.21 (0.83–5.89)
Lying on the ball carrier	0	N/A
Slipping/falling	3	105.88 (34.15–328.30)
Returning to feet	0	N/A
Bent at the knees Other	1 21	0.95 (0.13–6.74) 4.01 (2.61–6.15)
**Total**	**131**	**2.22 (1.87–2.63)**
**Tackler body position – tackler HIAs**		
Upright	55	1.89 (1.45–2.46)
Bent at waist	23	0.93 (0.62–1.40)
Diving	5	3.27 (1.36–7.86)
Leaping	3	3.78 (1.22–11.72)
Flopping	1	0.55 (0.08–3.90)
Lying on the ball carrier	0	N/A
Slipping/falling	0	N/A
Returning to feet	0	N/A
Bent at the knees	1	0.95 (0.13–6.74)
**Total**	**88**	**1.49 (1.21–1.84)**
**Tackler body position – BC HIAs**		
Upright	27	0.93 (0.64–1.36)
Bent at waist	2	0.08 (0.02–0.32)
Diving	0	N/A
Leaping	3	3.78 (1.22–11.72)
Flopping	2	1.10 (0.28–4.40)
Lying on the ball carrier	0	N/A
Slipping/falling	1	35.29 (4.97-250.54)
Returning to feet	0	N/A
Bent at the knees	0	N/A
**Total**	**35**	**0.59 (0.42–0.82)**
**BC body position – All HIAs**		
Upright	104	2.40 (1.98–2.91)
Bent at waist	7	0.79 (0.38–1.66)
Diving	15	7.15 (4.31–11.86)
Kicking	0	N/A
On the ground	2	2.08 (0.52–8.32)
Jumping	1	2.21 (0.31–15.69)
Bent at the knees	2	1.12 (0.28–4.48)
**Total**	**131**	**2.22 (1.87–2.63)**
**BC body position – tackler HIAs**		
Upright	75	1.73 (1.38–2.17)
Bent at waist	4	0.45 (0.17–1.20)
Diving	6	2.86 (1.28–6.37)
Kicking	0	N/A
On the ground	1	1.04 (0.15–7.38)
Jumping	0	N/A
Bent at the knees	2	1.12 (0.28–4.48)
**Total**	**88**	**1.49 (1.21–1.84)**
**BC body position – BC HIAs**		
Upright	27	0.62 (0.43–0.90)
Bent at waist	2	0.22 (0.06–0.88)
Diving	6	2.86 (1.28–6.37)
Kicking	0	N/A
On the ground	0	N/A
Jumping	0	N/A
Bent at the knees	0	N/A
**Total**	**35**	**0.59 (0.42–0.82)**

*Note* BC: ball carrier; HIA: head injury assessment; N/A: not applicable

### Ball Carrier Body Position

There were 35 ball carrier HIAs (26.7%), with an overall propensity of 0.59 (95% CI: 0.42 to 0.82) HIAs per 1,000 tackles (Table 1). An upright ball carrier sustained 27 HIAs (79.4%), with a propensity of 0.62 (95% CI: 0.43–0.90) HIAs per 1,000 upright ball carriers. The HIA propensity for bent-at-the-waist ball carriers was lowest at 0.22 (95% CI: 0.06–0.88) HIAs per 1,000 bent-at-the-waist ball carriers. A diving ball carrier had the highest propensity for an HIA to both ball carrier and tackler, with 7.15 (95% CI: 4.31–11.86) HIAs per 1,000 ball carrying dives, exceeding the propensities of the upright tackler and ball carrier, as shown in Table 1. The tackler sustained 88 HIAs, with 75 HIAs (85.2%) occurring when the ball carrier was in the upright position, with a propensity of 1.73 (95% CI: 1.38–2.17) HIAs per 1,000 per upright ball carries. The second lowest propensity for an HIA to the tackler occurred when ball carriers’ were bent at the knees, though only two HIAs occurred in such situations, with a propensity of 1.12 (95% CI: 0.28–4.48) HIAs per 1,000 tackles.

### Tackle Height

Propensities for HIAs associated with tackle height are presented in Table 2. A greater propensity for HIAs to the ball carrier was found when tackle height was at the head and neck compared to other tackle heights. A tackle with contact in the vicinity of the head/neck region accounted for 12.2% of all HIAs, with a propensity of 20.17 (95% CI: 12.36 to 32.92) HIAs per 1,000 tackles. The second highest propensity was observed with tackle contact made at the lower leg with 7.06 (95% CI: 2.65 to 18.81) HIAs per 1,000 tackles, although the total number of four (3%) suggests these are uncommon. Tackles at the height of the head/neck were 7.97 (95% CI: 4.66 to 13.63) times more likely to cause an HIA than tackles to the upper trunk, and 15.34 (95% CI: 7.95 to 29.61) times more likely to cause an HIA than tackles to the mid-trunk. Propensities for HIA from tackles to the mid trunk and lower trunk were statistically similar to one another (1.31 and 1.22 respectively), but significantly lower than tackles to the head/neck and upper trunk (Table 2).

**Table 2:** Propensity for HIAs for ball carriers and tacklers as a function of tackle height

Tackle height	HIA events	Propensity (HIAs per 1,000 events)
**Tackle height – all HIAs**		
Head/neck	16	20.17 (12.36–32.92)
Upper trunk	80	2.53 (2.03–3.15)
Mid trunk	20	1.31 (0.85–2.03)
Lower trunk	11	1.22 (0.68–2.2)
Upper leg	0	N/A
Lower leg	4	7.06 (2.65–18.81)
**Total**	**131**	**2.22 (1.87–2.63)**
(Above sternum)	96	2.96 (2.42–3.62)
(Below sternum)	35	1.31 (0.94–1.82)
**Tackler height – tackler HIA**		
Head/neck	0	N/A
Upper trunk	59	1.87 (1.45–2.41)
Mid trunk	18	1.18 (0.74–1.87)
Lower trunk	9	1.00 (0.52–1.92)
Upper leg	0	N/A
Lower leg	2	3.53 (0.88–14.11)
**Total**	**88**	**1.49 (1.21–1.84)**
(Above sternum)	59	1.82 (1.41–2.35)
(Below sternum)	29	1.09 (0.76–1.57)
**Tackler height – BC HIA**		
Head/neck	14	17.65 (10.45–29.80)
Upper trunk	19	0.60 (0.38–0.94)
Mid trunk	2	0.13 (0.03–0.52)
Lower trunk	0	N/A
Upper leg	0	N/A
Lower leg	0	N/A
**Total**	**35**	**0.59 (0.42–0.82)**
(Above sternum)	33	1.02 (0.73–1.43)
(Below sternum)	2	0.07 (0.02–0.28)

*Note* BC: ball carrier; HIA: head injury assessment

The specific propensity for tackler and ball carrier HIAs as a function of tackle height is also presented in Table 2. The tackler’s HIA risk, when tackling at the lower leg, was 3.53 (95% CI: 0.88–14.11) per 1,000 such tackles, however this tackle height is uncommon. The second highest HIA risk for the tackler involved the upper trunk, with a propensity of 1.87 (95% CI: 1.45 to 2.41) per 1,000 tackles. Broadly, tackling above the sternum saw the greatest risk with a propensity of 1.82 (95% CI: 1.41 to 2.35) per 1,000 tackles, as opposed to a propensity of 1.09 (95% CI: 0.76 to 1.57) per 1,000 tackles for tackles that were below the sternum.

The ball carrier is at a significantly increased risk when the tackle involves the head or neck, with a propensity of 17.65 (95% CI: 10.45–29.80) per 1,000 tackles. This was followed by tackles in the vicinity of the upper trunk, with a propensity of 0.60 (95% CI: 0.38–0.94) per 1,000 tackles. A low propensity for HIAs to the ball carrier occurred for tackles made in the mid trunk region with a propensity of 0.13 (95% CI: 0.03–0.52) per 1,000 tackles, with no HIAs recorded for tackles made in the region of the lower trunk, upper leg and lower leg.

### Ball Carrier Evasion Method

When the ball carrier did not utilise an evasion method, the HIA propensity for an HIA to either player was elevated, with a propensity of 3.73 (95% CI: 2.58 to 5.40) HIAs per 1,000 tackles (Table 3), compared to 1.38 (95% CI: 0.87 to 2.19) HIAs per 1,000 carries with footwork/step and 1.82 (95% CI: 1.03 to 3.20) HIAs per 1,000 carries with a twist-spin.

For the tackler specifically, when the ball carrier employs an evasion strategy by leading with the shoulder, tackler HIA propensity was greatest with 7.43 (95% CI: 2.79 to 19.80) HIAs per 1,000 tackles, followed by the ball bump with an HIA propensity of 3.72 (95% CI: 0.93 to 14.87) HIAs per 1,000 tackles and the forearm bumpers with an HIA propensity of 1.96 (95% CI: 1.43 to 2.68) per 1,000 tackles (Table 3). For the ball carrier, a lack of evasion method led to a propensity of 1.60 (95% CI: 0.91 to 2.82) HIAs occurring per 1,000 tackles.

**Table 3:** Propensity for HIAs as a function of the ball carriers’ evasion methods

Ball carrier evasion	HIA events	Propensity (HIAs per 1,000 events)
**BC evasion method – all HIAs**		
None	28	3.73 (2.58–5.40)
Hand fend	2	0.71 (0.18–2.84)
Forearm bumper	55	2.77 (2.13–3.61)
Shoulder	4	7.43 (2.79–19.80)
Ball bump	3	5.57 (1.80-17.27)
Lean/bend torso	6	1.13 (0.51–2.52)
Twist-spin	12	1.82 (1.03–3.20)
Side-on	0	N/A
Footwork/step	18	1.38 (0.87–2.19)
Ducked head	1	0.71 (0.10–5.04)
Jumping	1	2.35 (0.33–16.68)
Unknown	1	35.29 (4.97-250.54)
**Total**	**130**	**2.20 (1.85–2.61)**
**BC evasion method – tackler HIAs**		
None	11	1.47 (0.81–2.65)
Hand fend	1	0.36 (0.05–2.56)
Forearm bumper	39	1.96 (1.43–2.68)
Shoulder	4	7.43 (2.79–19.80)
Ball bump	2	3.72 (0.93–14.87)
Lean/bend torso	4	0.75 (0.28-2.00)
Twist-spin	11	1.67 (0.92–3.02)
Side-on	0	N/A
Footwork/step	15	1.15 (0.69–1.91)
Ducked head	0	N/A
Jumping	0	N/A
Unknown	1	35.29 (4.97-250.54)
**Total**	**88**	**1.49 (1.21–1.84)**
**BC evasion method – BC HIAs**		
None	12	1.60 (0.91–2.82)
Hand fend	1	0.36 (0.05–2.56)
Forearm bumper	16	0.81 (0.50–1.32)
Shoulder	0	N/A
Ball bump	1	1.86 (0.26–13.20)
Lean/bend torso	2	0.38 (0.10–1.52)
Twist-spin	1	0.15 (0.02–1.06)
Side-on	0	N/A
Footwork/step	1	0.08 (0.01–0.57)
Ducked head	1	0.71 (0.10–5.04)
Jumping	0	N/A
Unknown	0	N/A
**Total**	**35**	**0.59 (0.42–0.82)**

*Note* BC: ball carrier; HIA: head injury assessment; N/A: not applicable

### Tackler Head Position

For 53% of HIAs, the tackler’s head was positioned directly in front of the ball carrier, with a propensity of 9.23 (95% CI: 7.30 to 11.67) HIAs per 1,000 tackles (Table 4). This head position was 6.12 (95% CI: 4.15 to 9.03) times more likely to cause an HIA than if the tackler’s head was beside the ball carrier at contact, and 5.05 (95% CI: 3.04 to 8.39) times more likely to cause an HIA than if the tackler’s head was behind the ball carrier.

**Table 4:** Propensity for HIAs as a function of tackler’s head position

Tackler head position (relative to BC)	HIA events	Propensity (HIAs per 1,000 events)
In front	70	9.23 (7.30-11.67)
Beside	40	1.51 (1.11–2.06)
Behind	19	1.83 (1.17–2.87)
Above	2	0.82 (0.21–3.28)
**Total**	**131**	**2.79 (2.35–3.31)**

HIA: head injury assessment.

## Discussion

This study examined the tackle risk factors that result in an HIA in sub-elite (i.e., semi-professional) male rugby league players [1]. In this sample of sub-elite men’s rugby league players, the tackler is at a significantly greater risk of having an HIA than the ball carrier, consistent with previous findings [1, 5]. There was a 2.5-fold increased risk for the tackler compared to the ball carrier, similar in magnitude to that measured in elite level men’s rugby league and rugby union (NRL = 1.7 [1], and rugby union = 2.59 [5] fold increase for the tackler). The implication is that the risk mitigation strategies for sub-elite male rugby league, like those of elite male rugby league, should include a focus on modifying tackler behaviour into tackle, and not necessarily law change, which typically favours protection of the ball carrier against the tackler’s actions. That said, an important finding in this study, also consistent with previous research, is that tackles at the height of the head/neck result in a significantly greater likelihood of causing an HIA overall, with particularly high risk to the ball carrier. Tackle behaviour modification may be achieved in a multitude of ways. A reduction in tackle height by the governing bodies may reduce the prevalence of upright, high tackles, and/or tackle education strategies to modify player pre-tackle behaviour before they enter into contact may be of critical importance.

The overall HIA risk is 20.17 HIAs per 1,000 head/neck tackles, which is 7.95 fold greater than for upper trunk tackles and 15.4 times greater than for mid-trunk tackles. This risk is particularly pronounced for the ball carrier, with a ball carrier HIA risk of 17.65 HIAs per 1,000 head/neck tackles. Indeed, 33 of the 35 ball carrier HIAs (94.3%) occurred as a result of tackles above the sternum, and so ball carrier HIAs in sub-elite rugby league occur almost exclusively as a result of tackles that are either illegal (head/neck) or very high (upper trunk). In essence, the focus is to mitigate the risk of the outcome (i.e., that players’ heads are near each other or near the upper trunk at the time of contact in the tackle). This suggests that law change or law application, but also modifying players’ behaviour entering into the tackle, might significantly lower the risk to the ball carrier, and overall.

Tackle height, and thus head contact or proximity at contact, is to some degree related to the body position adopted by players in the tackle. When the tackler adopts an upright body position at contact, HIA risk is significantly increased compared to bent tacklers [1, 5]. In the present study, a 2.75 fold increase in overall risk (tackler and ball carrier) was found for an upright compared to bent tackler at contact, and that the tackler HIA risk is also greater when upright with a 2.0-fold higher risk compared to the bent tackler. A similar result was reported in the NRL, where a 3.2-fold higher overall risk for an HIA was reported for upright vs. bent tacklers [1], and in professional rugby union with a 1.5-fold increase in risk when tacklers were upright compared to bent-at-the-waist [1, 5]. Upright tacklers are more vulnerable to head impact events compared with tacklers adopting a bent-at-the-waist body position [1, 5].

An upright tackler body position at contact also created the greatest risk to the ball carrier, with 27 of the 35 ball carrier injuries occurring when the tackler was upright, with a significantly higher propensity (0.93 per 1,000 tackles), than when the tackler was bent (0.08 per 1,000 tackles). This is likely related to the finding discussed above, that head/neck and upper trunk tackles are significantly more likely to injure the ball carrier, because these higher contacts are more likely when the tackler is in an upright position. Previous studies involving elite rugby league players [4] show that risk mitigation that removes a player’s head from high-propensity locations at contact that occur from tackling too high or upright may contribute to an overall reduction in concussion risk. Change or modification of existing tackle laws regarding the height of the tackle have also been explored in studies involving elite rugby union [11]. It has been found that lowering of the permitted height of the tackler’s contact area may reduce the risk of concussion to both ball carrier and tackler, with the greatest reductions in risk being to the ball carrier [6, 11].

Because the upright and the bent-at-the-waist tackler are the two most commonly adopted body positions across all tackles, accounting for 84% of all tackles which result in HIAs, the relative risks involved with these two key tackle techniques are important when considering injury risk reduction programs that focus on modifying tackling behaviour for both the tackler and the ball carrier. Adopting this body position is the most effective method of decreasing the speed of the play-the-ball and preventing the off-load [12]. We confirm that when either or both the tackler and the ball carrier are upright, they are at increased risk of HIAs. Whilst some tackle behaviours were not found to significantly increase risk for an HIA, there is a distinct implication, consistent with the findings in the NRL and elite-level rugby union research [1, 13], that there clearly is a lower risk for an HIA when the ball carrier and tackler adopt the bent position.

Other tackle body positions with high propensities for HIAs were identified. One of these risks involved either the ball carrier or the tackler slipping or falling into contact. The likelihood of mitigating these types of risks appears to be low, given the unpredictable nature of a player slipping/falling. Another high propensity risk occurred when either the tackler or the ball carrier dives (often when attempting to score or prevent a try, or when a tackler is attempting to make a cover tackle). The prospect of successfully implementing a risk mitigation strategy for these types of game play situations is also considered to be quite low, because it is a well-established principle that the successful implementation of any injury risk mitigation strategy must also take into consideration the effect that any strategy may have on performance [6]. A tackler diving to make a tackle and the ball carrier diving to score a try are critical performance-based game play situations, and changes to these through law or education would be unlikely to be adopted by coaches and players. In addition, although the propensity is high for both situations, the prevalence of these situations is low, suggesting that even a successful mitigation strategy would have a small real-world impact of reducing the risk [15].

The increased risk of head/neck and upper trunk tackles for upright players suggests that the QRL may consider implementing changes that could mitigate the risk of contact with the head by modifying players’ tackling behaviours [12]. This may include the application of evidence-based tackle re-education coaching programs underpinned by this study’s scientific evidence. Although there is limited evidence to support the effectiveness of prevention or concussion education programs [14], an education program that is focused on teaching the tackler to engage in contact with the ball carrier’s lower or mid-trunk regions, areas that had a low propensity for HIA in the present study, may be effective [15].

Given the similarities in these findings with those at the elite level [1, 5], the risk mitigation strategies employed at the elite male level may translate effectively to the sub-elite male level. Historically, interventions aimed at reducing the tackle height (i.e., reducing the incidence of those tackles that involve contact to the head, neck, and shoulder) have achieved varied results. Law change to move the legal height of the tackle to the level of the armpit did not reduce overall concussion risk, and in fact increased risk to tacklers in one study in English elite rugby union [12]. In another study [16], the risk only began to decrease in the latter part of a similar law change trial, suggesting that time for adaptation and effectiveness is required, and that the study in elite English rugby union players may not have provided sufficient time for behaviour change for coach and player adaptation. In that study, the trial was also implemented mid-season for a short period in a different competition before players reverted back to normal tackle height to complete the season. This short, disruptive adjustment may explain why tackler risk increased.

Rugby league governing bodies may look to continue to explore options for encouraging the tackler to be bent-at-the-waist and knees, but correct implementation is crucial. Studies relating to tackle characteristics and injury risk informed the rationale for World Rugby’s implementation of risk mitigation strategies in the elite game, where the legal tackle height has remained unchanged, but tougher sanctions were introduced for breaches of the previous tackle height rule [17]. The intention is that sanction will drive behaviour change to avoid the risk of punitive red cards, with the result that players will target lower contact on their opponent’s bodies, thus avoiding the head. The effectiveness of this approach to date may have been compromised by inconsistency of application by match officials, and potentially also the relative infrequency of cards and sanction to act as an effective deterrent that would drive behaviour change. To improve the likelihood for success of World Rugby’s ‘tackle re-education’ [19] risk mitigation strategy, a constraints-led approach, that considers the interaction of the multiple components of tackling (i.e., task), the environment, and the individuals (i.e., interaction of the ball carrier and tackler(s)), might be preferred. Implementing the Behaviour Change Wheel approach (i.e., the four behaviour science tools - capability, opportunity, motivation, behaviour model), might help facilitate tackle the behaviour change and maintain the new tackling behaviour over time [18].

Another important tackle-based risk factor that was evaluated in the current study was to examine how the ball carrier’s method of evasion influenced the risk of an HIA for the ball carrier and/or the tackler. The results revealed that contact avoiding methods, including using footwork to rapidly decelerate one to two steps before contact, significantly lowered the overall HIA risk than methods where the ball carrier does not decelerate and seeks contact through bumping the opponent with the ball or a body part, or using no evasion method. This suggests that a more evasive game (i.e., modifying player behaviour before the time of contact) would reduce injury risk, in addition to an improved performance outcome (e.g., line break/swift play-the-ball). Whilst ball carrier evasion techniques significantly lower HIA risk to both the ball carrier and the tackler in rugby league, these findings are not necessarily consistent across sporting codes with studies in rugby union suggesting ball carrier evasion may increase the injury risk to the tackler [12]. Speed and continuity of match-play in rugby league may be a contributing factor to the difference observed between the two rugby codes.

There is potential to reduce the frequency of this tackle-based risk factor via coaching re-education of tacklers to keep their heads outside ball carriers’ bodies and/or make contact with their chest/pectoral region, such as a previously described tackle technique [19]. This technique differs from the traditional smother NRL tackle technique as the tackler adopts a partially bent over posture (versus upright), contacts the ball carrier in the mid/upper torso (i.e., base of chest versus upper torso), and positions their head outside the ball carrier shoulder (compared to inside the ball carrier’s shoulder width) [19]. This change in tackle behaviour before contact may allow the tackler to reduce the HIA risk, even when executing a smother (i.e., over the ball) tackle technique, because it avoids the elbow-to-head contact scenario at contact while adopting a partially bent-at-waist-tackle posture and contacting the ball carrier in the mid/upper torso, all lower risk scenarios in the present study. Further behaviour modification technique interventions may be focused on the ball carrier, to introduce evasion techniques such as footwork to decelerate prior to contact that reduce the risk of head contact. Such interventions may also see a potential reduction in the contact forces involved and improved tackle performance whereby both players can be more reactive to evade or/tackle their opponent effectively.

### Limitations

There are a number of limitations with the current study. First, this study has a relatively small sample size, with greater variances and higher propensity than that observed in the NRL study [1]. Second, the results coded were obtained from one season only, as opposed to multiple seasons. Third, the results were coded by a single rater, although this was consistent with previous protocols utilised in the NRL study [1]. Fourth, this study was conducted with men and does not necessarily translate to the women’s game. The different physiological characteristics of male and female players may create different risk factors because of variations in force application and tolerance. The level of competition seen in male sub-elite rugby league may not translate to lower levels of play or ages, making generalisation of the results to these levels of play limited.

## Conclusions

The tackler had a significantly greater risk of sustaining an HIA compared to the ball carrier. This risk is greatest when both players are in the upright position at the point of contact. The risk for an HIA was lowest when both the tackler and ball carrier are bent in the tackle, which is consistent with the findings suggesting that the HIA risk to both players is greatest when the tackle height is in the region of the head and neck and upper trunk. The other circumstance where the tackler is vulnerable to an HIA is when the ball carrier utilises his forearms to bump or fend the tackler. The implication of these results is that potential risk mitigation strategies could focus on the reduction of higher-risk tackles in the region of the upper trunk and above, as well as limiting forearm use by the ball carrier, either through law change or education, or a combination of both of these strategies.

### How may this Influence Future Practice?

The tackle characteristics associated with head impacts leading to HIAs were similar in this sample of men participating in semi-elite level of rugby league compared to prior studies with men in professional rugby union [5] and rugby league [1]. This study provides some evidence to support the rationale for intervention strategies designed to reduce the frequency of head impacts and HIAs. Risk mitigation strategies may include the re-evaluation of coaching methods and potential rule modifications aimed at reducing the frequency of upright tackles. Further exploration of the ball carrier’s evasive techniques and the risk for HIEs to the tackler are warranted.

## Data Availability

The datasets presented in this article are not readily available because of privacy considerations. The original contributions presented in the study are included in the article, further inquiries can be directed to the corresponding author (Andrew Gardner, andrew.gardner@sydney.edu.au).

## Abbreviations

HIA: Head Injury Assessment
HIE: Head Injury Event
QRL: Queensland Rugby League
NRL: National Rugby League
